# Evaluating smartphone-based cough frequency monitoring for tuberculosis screening and triage in Uganda: A mixed-methods evaluation

**DOI:** 10.1371/journal.pgph.0005201

**Published:** 2026-10-01

**Authors:** Patrick Biché, Francis Kayondo, Annet Nalutaaya, James Mukiibi, Mariam Nantale, Joowhan Sung, David W. Dowdy, Achilles Katamba, Emily A. Kendall

**Affiliations:** 1 Department of Epidemiology, Johns Hopkins Bloomberg School of Public Health, Baltimore, Maryland, United States of America; 2 Uganda Tuberculosis Implementation Research Consortium, Walimu, Kampala, Uganda; 3 Division of Infectious Diseases, Johns Hopkins University School of Medicine, Baltimore, Maryland, United States of America; 4 Clinical Epidemiology Unit, Department of Medicine, Makerere University College of Health Sciences, Kampala, Uganda; Centro de Investigacao e Treino em Saude da Polana Canico, Instituto Nacional de Saude, MOZAMBIQUE

## Abstract

Symptom-based tuberculosis screening usually depends on self-reported cough, which is subjective. Passive smartphone cough monitoring offers a more objective alternative. This study assessed the diagnostic accuracy and implementation feasibility of smartphone-based cough frequency monitoring for identifying likely TB cases in Uganda. Adults (≥15 years) screened in community settings or tested at Kampala health facilities underwent microbiological testing and 48 hours of cough monitoring with the Hyfe Research app. TB status was determined by Xpert MTB/RIF Ultra and culture, with inverse probability weighting to adjust for differential enrollment. Cough frequency was compared between participants with and without TB, and accuracy assessed using weighted ROC curves. Staff interviews, analyzed thematically, explored provider-side implementation challenges. Of 884 enrolled participants, 197 had both valid recordings and TB status (101 community, 96 facility). Median cough frequency was higher among those with TB: 2.2 coughs/hour (IQR 0.8–6.1) vs 0.9 (0.4–2.0) in the community, and 6.7 (2.6–27.5) vs 2.4 (0.9–4.8) in facilities (Wilcoxon P < 0.0001 for both). AUCs were 0.69 (95% CI 0.58–0.79) in the community and 0.76 (95% CI 0.60–0.88) in facilities. For self-reported cough at 90% sensitivity, specificities were 55% (95% CI 29–88%) and 36% (95% CI 17–68%), respectively. Recorded cough frequency correlated moderately with self-reported cough severity, Saint George’s Respiratory Questionnaire scores, and staff-observed coughs. Staff cited device visibility, stigma, and security concerns as barriers. Smartphone-recorded cough frequency was associated with TB status but did not meet accuracy thresholds for stand-alone screening or triage, and implementation problems limited data collection. Addressing these operational barriers will be critical to future development and deployment of cough monitoring tools for TB screening.

## Introduction

Decisions about whether to test for tuberculosis (TB) are often guided by symptoms, particularly cough, despite evidence that, during population-based screening, approximately half of individuals with microbiologically confirmed TB screen negative for symptoms [1, 2]. However, cough may be unrecognized, unreported, or assessed inconsistently by different individuals [3]. This unreliability of subjective symptom reporting presents challenges for healthcare providers and TB screening programs. More objective cough measurement tools that improve symptom assessment may therefore enhance the performance of TB screening and triage strategies.

Tools to quantify cough have evolved over decades, progressing from mechanical counters and symptom diaries to digital applications that leverage artificial intelligence (AI) to identify, count, and characterize cough sounds [4]. Recent advancements in digital acoustic surveillance have further expanded these capabilities, with AI models now demonstrating promise in detecting disease-specific acoustic patterns [5]. Digital cough monitoring approaches can be broadly categorized as those that measure cough frequency (counting the number of coughs over time) and those that analyze the acoustic signature of sounds to identify disease-specific patterns. This study focuses on cough frequency monitoring. The use of cough-based diagnostic tools expanded during the COVID-19 pandemic, reflecting growing interest in passive, digital respiratory surveillance [5]. The Hyfe Research App (“Hyfe”) is one such tool for which early studies demonstrated high accuracy in detecting cough sounds in a controlled setting [6–8]. However, the application of these tools for infectious disease screening, especially in real-world, resource-constrained settings, remains underexplored.

Digital cough measurement tools have potential to improve TB screening and triage in high-TB-burden, resource-limited settings where TB diagnostic capacity is constrained but digital access is increasing. However, evidence remains limited about their feasibility and diagnostic utility in these settings. This study evaluates whether cough frequency, measured via a smartphone-based passive monitoring app, can distinguish individuals with TB in both community-based screening and health facility-based triage settings, evaluating accuracy against an established target product profile (TPP) from the World Health Organization (WHO) [9,10]. We also interviewed staff to understand perceptions of cough recording and potential obstacles to its broader implementation.

## Methods

### Study population

This analysis includes individuals who participated in either community-based screening or routine clinic-based diagnosis for presumptive TB in the Kampala, Uganda, metropolitan area between 17 March 2021 and 06 December 2024. This study was embedded within parent studies that enrolled participants who had been tested with Xpert Ultra as a symptom-agnostic screening test within high-risk communities (the “community” cohort) or as a diagnostic test during (primarily symptom-driven) routine diagnostic evaluation in ambulatory health facilities (the “health facility” cohort) [11,12]. Both cohorts recruited individuals aged ≥15 years.

Both cohorts recruited all individuals with trace Ultra results during the enrollment period, as well as a selection of individuals with positive (greater than trace) or negative Ultra results. For groups more prevalent than those with trace results (those with negative results in the community setting, and those with both positive-greater-than-trace and negative results in the health facility setting), we matched by age, sex, and human immunodeficiency virus (HIV) status to those with trace results; individuals with positive-greater-than-trace results in the community setting were enrolled universally until a target sample size was reached. Enrolled participants from both cohorts were offered additional *M. tuberculosis* microbiological testing and smartphone app-based cough frequency measurement. Further details of the parent studies are described elsewhere [11,12].

### Data collection

At the time of enrollment into both cohorts, participants underwent standardized interviews that included demographic information, symptom characterization, and TB treatment history. Participants screened in the community after July 2021 were also asked, at the time of screening, whether they currently had a cough. Symptoms were characterized through a questionnaire about the presence and duration of individual symptoms as well as the Saint George’s Respiratory Questionnaire (SGRQ) [13] (designed to measure health-related quality of life in chronic pulmonary disease), a cough severity rating (self-assessed using a visual analogue scale from 0 to 100, set to zero for those not reporting cough [14]), and observations of cough by research staff during the enrollment (assessed as both a qualitative assessment and a five-minute count of coughs). The applicable subset of interview questions can be found in Appendix A in S1 Text. Additional testing included solid and liquid cultures, a repeat Ultra test on expectorated sputum, and a posteroanterior chest x-ray (CXR).

Participants were also asked to complete 48 hours of cough frequency recording following the enrollment visit. Consenting participants were provided with a study smartphone equipped with the Hyfe research app, a lapel microphone, and an external battery bank for charging. All equipment was procured by the study team and was not provided or subsidized by Hyfe Inc. This app processes sound continuously and saves a < 0.5 second recording whenever there is an “explosive” sound that exceeds background noise signal by a set threshold. Each of these sounds then undergoes AI-based analysis and is assigned a score between 0 and 1, correlated to the probability of being a cough. The Hyfe app has demonstrated high accuracy in detecting cough sounds in controlled settings, with reported sensitivity exceeding 90% and specificity above 95% for identifying coughs among ambient sounds [7,8]. Participants were asked to keep the phone near them for 48 hours. They were also provided with a lavalier microphone, which they were asked to connect and wear whenever possible (to better differentiate sounds coming from near their own mouth) and a spare battery pack (for recharging if electricity was unavailable).

To better understand barriers to cough data collection, interviews were conducted with research staff who had used the Hyfe research app to measure cough frequency. Participating staff were asked about feedback they received from participants, factors they considered when deciding to give the device to participants, and questions related to the recording process and areas for improvement. Interviews were audio-recorded, transcribed verbatim, and analyzed using thematic analysis in NVivo software [15]. The interview guide is listed in Appendix B in S1 Text.

### TB status classification

In classifying participants’ TB status for diagnostic accuracy analyses, we considered Ultra and culture results. For participants with a history of TB treatment (for whom Ultra false-positive results are more likely), we considered culture results to be definitive [16]. Absent a history of TB treatment, those with a positive Ultra results greater than trace were classified as having TB, but those with an initial trace result required at least one additional trace result, or any other positive Ultra or culture result, to be classified as having TB. Those with a negative initial Ultra result and no prior treatment required another negative Ultra or culture result (and no positive results) to be classified as TB-negative and were excluded otherwise. As a secondary analysis, diagnostic accuracy was also assessed against a culture-based microbiological reference standard (MRS), restricted to participants with available culture results.

### Statistical analysis

We applied a developer-recommended score cutoff of ≥0.85 to define a cough among device-captured explosive sounds. To verify device proximity, participants were asked upon device return to indicate any periods when the device was more than two meters away. We limited analysis to time periods when (1) the participant’s recording app was active by the device timestamp log, (2) the time period feel within the window when research staff documented that the participant had the device, and (3) the participant did not report having been more than two meters away from the device. We then calculated a mean cough frequency in coughs per hour for each participant.

Because individuals with different results (negative, trace, positive) on an initial Ultra test result that prompted enrollment into the parent study had different probabilities of recruitment into the parent study, analyses of diagnostic accuracy were weighted to reflect the original population that had undergone screening or diagnostic testing. We determined the proportion of individuals with each Ultra result among all participants in our community-based screening intervention, and (separately) in a tally of 12 months of Ultra test results from patients evaluated for presumptive TB at five participating health facilities. We then compared these proportions to the proportion of each Ultra result type among participants who completed cough recording, and we weighted the data by this relative prevalence using inverse probability of selection weight (Table A in S1 Text). All diagnostic accuracy analyses were performed separately for community-based screening versus health facility-based diagnostic testing. Values for weighting can be found in. Cough frequency comparisons were not conducted as a matched analysis, and demographic differences between Xpert result groups were not accounted for in the inverse-probability weights.

We compared cough frequency by TB status using weighted Wilcoxon rank sum tests. We also examined correlations between cough frequency and cough severity (visual analogue scale), Saint George’s Respiratory Questionnaire scores, and staff-observed cough counts measures using Spearman correlation coefficients. We estimated the accuracy of cough frequency for classifying TB status by creating weighted receiver operating characteristic (ROC) curves and estimating areas under the curve (AUCs) with 95% confidence intervals (WeightedROC package version 2020.1.0.3) [17]. Sensitivity and specificity of subjective cough screening were calculated among participants with confirmed TB status who had undergone symptom screening at the time of initial Ultra testing in the community, with weighted binomial 95% confidence intervals. To compare screening accuracy between self-reported cough (a binary response to “Do you have cough?” asked during community screening) and measured cough frequency, we used ROC analysis to identify the cough frequency threshold (mean cough frequency in coughs per hour) that yield equivalent specificity to reported cough, and the sensitivity of that threshold was statistically compared to the sensitivity of reported cough using McNemar’s test for paired proportions. Diagnostic accuracy was benchmarked against both the 2014 and 2025 WHO TPP thresholds for TB screening tests. The 2014 WHO TPP specifies minimum targets of ≥90% sensitivity and ≥70% specificity [9]. The 2025 WHO TPP distinguishes between Type 2 (high-sensitivity screening) profiles with minimal targets of ≥90% sensitivity and ≥60% specificity, and optimal targets of ≥95% sensitivity and ≥85% specificity and Type 3 (high-specificity triage) profiles, with minimal targets of ≥60% sensitivity and ≥98% specificity [10]. Cough frequency thresholds required to meet each TPP profile are reported in Table C in S1 Text. All statistical analyses were conducted using R version 4.5.0 [18]. Secondary analyses used culture positivity alone as the reference standard (MRS), applying the same weighted ROC and bootstrap CI approach, and results were benchmarked against the same WHO TPP thresholds.

### Ethics statement

Written informed consent was obtained from all adult study participants (aged ≥18 years). For participants aged 15–17 years, written adolescent assent was obtained along with written parental or guardian informed consent. Additional information regarding the ethical, cultural, and scientific considerations specific to inclusivity in global research is included in the Supporting Information (S1 Checklist). The study was approved by the Institutional Review Boards at the Johns Hopkins University School of Medicine and Makerere University School of Public Health.

## Results

### Completion of cough recording

A total of 400 community and 484 health facility participants were enrolled during the analysis period, of whom 147 (36%) community and 126 (26%) health facility participants received a device and completed at least one hour of cough frequency recording (Fig 1). In addition, 19 participants received a device but failed to complete at least one hour of recording (12 community and seven health facility). No devices were available for six participants. When devices were available, the reason for not receiving a device was documented for 209 participants (documented only for the first eight months of the study): 110 (53%) were deemed ineligible by study staff, mainly due to safety concerns, and 99 (47%) declined by participants for reasons that included feeling unsafe carrying the device or objections of partners/family. After also excluding 76 participants with missing cough data or indeterminate TB status, 101 (25%) participants enrolled from community screening and 96 (20%) enrolled from health facility-based evaluation were included in this analysis. Of the 101 community cohort participants included in the cough frequency analysis, 56 had been asked about symptoms at the time of screening, as symptom assessment was only introduced in the community screening protocol after July 2021. A total of 8625 hours of cough recording from these participants were analyzed, with a median recorded time of 45 hours (interquartile range [IQR] 27–49) per participant.

**Fig 1:**
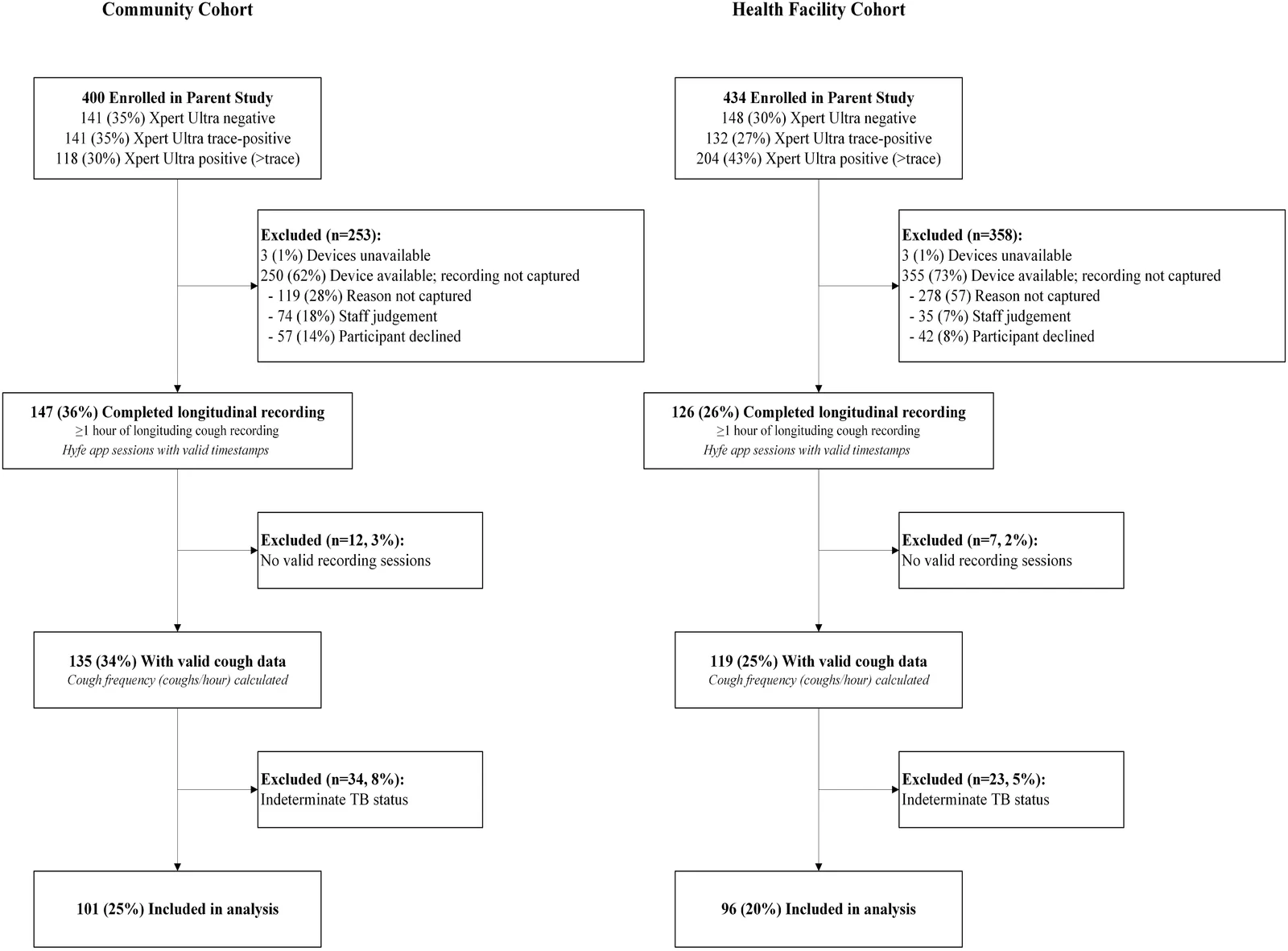
Flowchart of study participants and reasons for exclusion from analysis. Shown are the results of recruitment and enrollment into the present analysis of smartphone-based cough recording, relative to sampling in the parent study. “Reason not captured” for lack of completing longitudinal recording reflects the parent study design, in that only solicited cough sounds were recorded for some participants resulting in no documented reason for not completing longitudinal recording. No valid sessions occur when there are no recorded sessions that fall within the period research staff reported the participant had the device. Other reasons may include device malfunction, improper use of devices, or data loss.

### Participants

Included participants were 53% male with a median age of 32 (IQR 25–38) (Table 1). Overall, 27% were living with HIV (11% in the community cohort and 44% in the health facility cohort), 19% had a history of previous TB, and 21% reported current or former smoking. In the community cohort of 56 participants who were asked about symptoms at the time of screening, 24 (43%) reported having a cough. In the health facility cohort, cough was assessed only at enrollment, after initial Ultra test results, with 85% (82/96) of participants reporting cough. Among those from either cohort who reported cough at study enrollment, the median self-reported severity was 25 (IQR 8–60) on a scale of 0–100 and the median duration was 4 weeks (IQR 3–10). CXR was abnormal in 46% of participants.

**Table 1:** Characteristics of participants included in cough frequency analyses.

Recruitment Source	Overall	Community		Health Facility	
**TB Status**	**Negative**	**Positive**	**Negative**	**Positive**
**N**	197	54	47	68	28
**Total hours recorded**	8625	2323	2281	2902	1119
**Median hours recorded (IQR)**	45 (27–49)	45 (30–48)	48 (27–53)	44 (26–48)	46 (33–50)
**Male, n (%)**	104 (53)	30 (56)	31 (66)	29 (43)	14 (50)
**Age, median (IQR)**	32 (25 –38)	28.5 (24–35)	32 (24–37.5)	35 (27–40.25)	30 (23–36.5)
**HIV positive, n (%)**	53 (27)	7 (13)	4 (9)	32 (47)	10 (36)
**Previous TB, n (%)**	38 (19)	7 (13)	0 (0)	29 (43)	2 (7)
**Smoker, n (%)**	41 (21)	6 (11)	15 (32)	13 (19)	7 (25)
**Reported cough** ^*^ **, n (%)**	106/152 (70%)	12/29 (41)	12/27 (44)	55 (81)	27 (96)
**Cough severity, median (IQR)**	25 (8–6)	0 (0–10)	40 (20–60)	32 (10–50)	50 (30–70)
**Cough duration in weeks, median (IQR)**	4 (3–10)	2 (1–4)	4 (2–12)	3 (2–7)	4 (2–10)
**SGRQ** ^**†**^ **, median (IQR)**	15 (6–44)	10 (6–20)	22 (12–65)	15 (6–49)	34 (26–53)
**Ultra>trace, n (%)**	49 (25)	0 (0)	28 (60)	0 (0)	21 (75)
**Ultra trace, n (%)**	11 (6)	0 (0)	4 (9)	3 (4)	4 (14)
**Culture positive, n (%)**	55/190 (29)	0/52 (0)	30/46 (65)	0/64 (0)	25/28 (89)
**Abnormal CXR** ^**‡**^ **, n (%)**	91/185 (49)	11/53 (21)	36/45 (80)	24/64 (38)	20/23 (87)

*Reported during screening in the community cohort and during enrollment in the health facility cohort.

† Saint George’s Respiratory Questionnaire, with higher scores (on a scale from 0 to 100) representing greater impairment in respiratory-related quality of life.

‡ Chest x-ray.

### Cough frequency

In the community cohort, participants with TB had higher weighted median cough frequency on enrollment than TB-negative participants (2.2 coughs per hour (IQR 0.8–6.1) vs 0.9 coughs per hour (IQR 0.4–2.0), *P* < 0.0001; Fig 2a). A similar pattern held in the health facility cohort, with a weighted median of 6.7 (IQR 2.6–27.5) vs 2.4 (IQR 0.9–4.8) coughs per hour among participants with versus without TB (*P* < 0.0001; Fig 2b).

**Fig 2:**
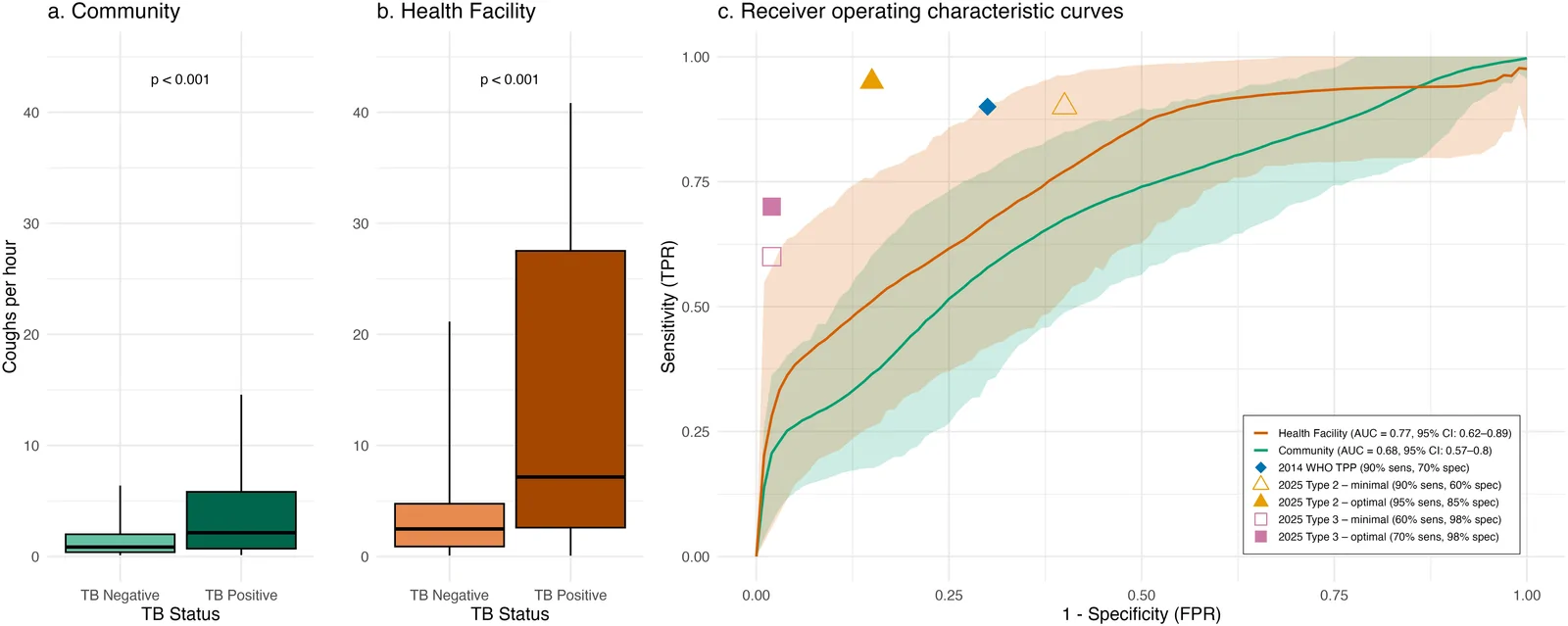
Relationship between weighted cough frequency and TB status, stratified by enrollment location. Boxplots show the distribution of measured cough frequency by TB status among community participants (panel a) and health facility participants (panel **b)**. The central line within each box represents the median; the lower and upper edges of the box correspond to the 25^th^ (Q1) and 75^th^ (Q3) percentiles, respectively; and whiskers extend to the smallest and largest values. Panel c shows weighted receiver operating characteristic curves for cough frequency as a predictor of TB in both community (green, AUC = 0.68, 95% CI: 0.57–0.80) and health facility settings (orange, AUC = 0.77, 95% CI:0.62–0.89). Shaded areas represent 95% confidence intervals. Reference points indicate target product profiles for TB screening tests: the 2014 WHO target product profile (90% sensitivity, 70% specificity); the 2025 Type 2 minimal benchmark (90% sensitivity, 60% specificity); the 2025 Type 2 optimal benchmark (95% sensitivity, 85% specificity); the 2025 Type 3 minimal benchmark (60% sensitivity, 98% specificity); and the 2025 Type 3 optimal benchmark (70% sensitivity, 98% specificity).

Recorded cough frequency had an AUC of 0.69 (95% CI, 0.58–0.79) in the community and 0.76 (95% CI, 0.60–0.88) in the health facility (Fig 2c, Table 2). Benchmarked against both the 2014 WHO TPP and the 2025 WHO TPP Type 2 and Type 3 profiles, recorded cough frequency did not meet sensitivity-specificity targets in either the community or health facility cohort (Fig 2c, Table C in S1 Text). This was lower than the AUC for self-reported cough in the community (0.83 [95% CI, 0.75–0.90]), but higher than in the health facility (0.63 [95% CI, 0.57–0.68]). Among community participants who completed cough recording, a cutoff of 1.51 coughs per hour achieved equivalent specificity to self-reported cough screening (59%) but significantly higher sensitivity (63% vs. 44%; *P* = 0.04).

**Table 2:** Diagnostic performance of recorded cough frequency and self-reported cough for TB detection, by enrollment location.

Measure	Metric	Community (95% CI)	Health Facility (95% CI)
Recorded cough frequency	AUC	0.68 (0.57–0.80)	0.77 (0.62–0.89)
Self-reported cough	AUC	0.87 (0.79–0.94)	0.75 (0.62–0.86)
Recorded cough frequency	Sp at 90% Se	16% (6%–47%)	47% (11%–67%)
Self-reported cough	Sp at 90% Se	55% (29%–88%)	36% (17%–68%)
Recorded cough frequency	Se at 70% Sp	59% (35%–77%	67% (42%–91%)
Self-reported cough	Se at 70% Sp	87% (76%–96%	64% (40%–88%)

Sp = Specificity, Se = Sensitivity.

In a secondary analysis using culture alone as the reference standard, 27 culture-positive and 70 culture-negative community participants and 24 culture-positive and 64 culture-negative health facility participants had valid cough recordings and culture results. Participants with culture-confirmed TB had higher weighted median cough frequency than culture-negative participants in both cohorts (community: 3.4 [IQR, 1.7–7.0] vs. 0.8 [IQR, 0.4–2.0] coughs per hour, *P* < .0001; health facility: 7.2 [IQR, 2.6–27.5] vs. 2.4 [IQR, 0.9–4.6] coughs per hour, *P* < .0001). The AUC for recorded cough frequency against the culture MRS was 0.78 (95% CI, 0.64–0.88) in the community and 0.78 (95% CI, 0.63–0.9) in health facilities, consistent with the primary analysis.

Recorded cough frequency was positively correlated with several subjective and observed cough measures, with variation by enrollment location (Fig 3, Table 3). In the community, the strongest correlation was with cough severity (ρ = 0.56, p < 0.001), followed by interview-observed coughs (ρ = 0.43, p < 0.001) and SGRQ score (ρ = 0.37, p = 0.033). In the health facility, interview-observed coughs showed the strongest association (ρ = 0.48, p < 0.001), with weaker but significant correlations for cough severity (ρ = 0.32, p = 0.0017) and coughs observed in 5 minutes (ρ = 0.33, p = 0.0014). Cough duration at enrollment was not significantly correlated with recorded cough frequency in the health facility setting (ρ = 0.14, p = 0.21), and cough duration at screening was not significantly correlated in the community (ρ = 0.18, p = 0.41).

**Table 3:** Spearman correlations between recorded cough frequency and subjective or observed cough measures, by enrollment location.

Cough Measure	Community ρ	Community p	Health Facility ρ	Health Facility p
Cough duration at enrollment	0.27	0.039	0.14	0.21
Cough duration at screening	0.18	0.41	—	—
Cough severity	0.56	<0.001	0.32	0.0017
SGRQ score	0.37	0.033	0.23	0.046
Coughs observed in 5 minutes	0.28	0.005	0.33	0.0014
Coughs observed during interview	0.43	<0.001	0.48	<0.001

**Fig 3:**
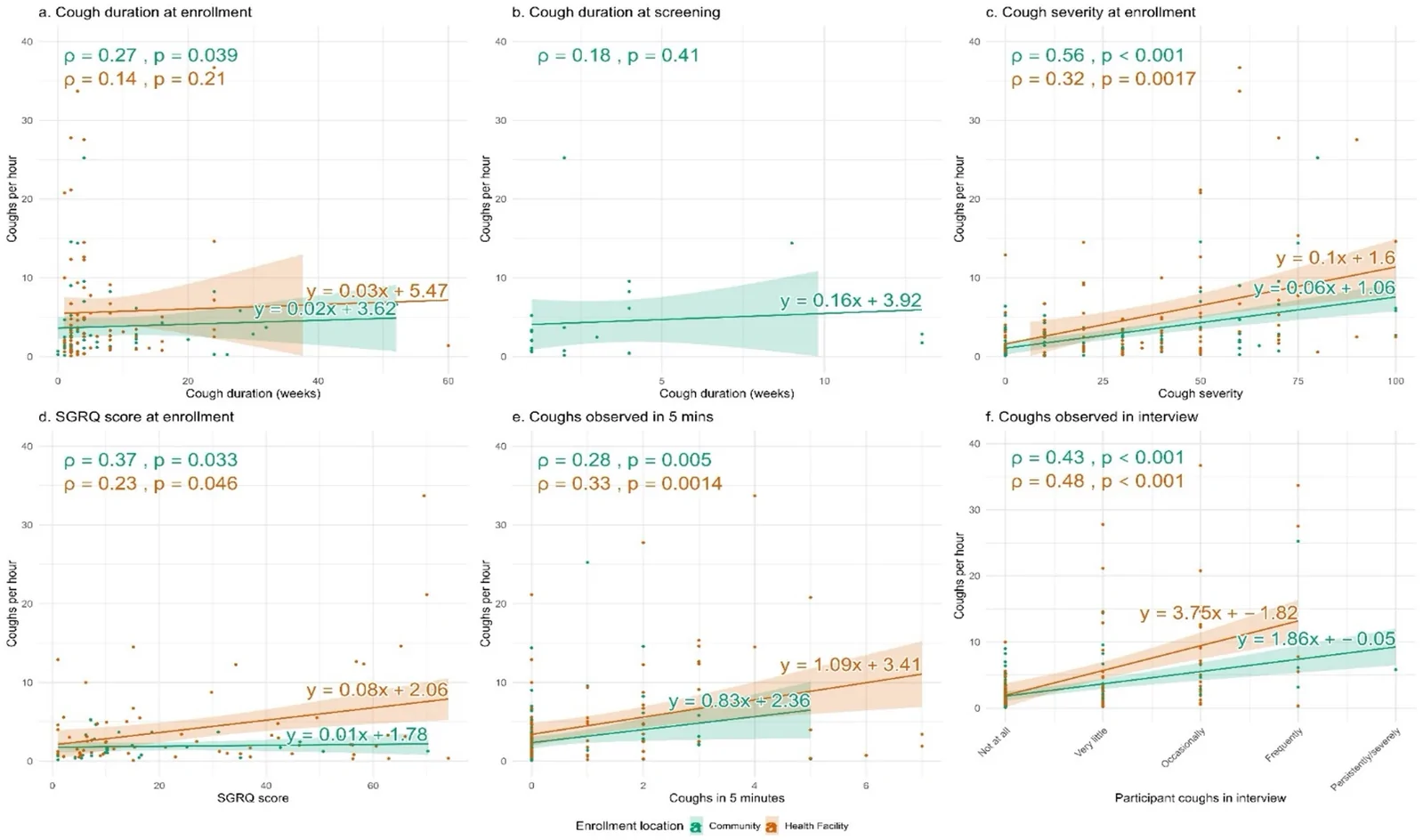
Associations between Hyfe app recorded cough frequency and participant-recorded or observed indicators. Each plot includes estimated regression lines with 95% confidence intervals. Green points and lines represent community participants; orange represents health facility participants. Spearman correlation coefficients (ρ) and p-values are provided for each group. SGRQ: Saint George’s Respiratory Questionnaire.

### Staff feedback

Eight research staff were interviewed about their experience collecting cough-recording data. They described logistical and social barriers that resulted in low completion rates (Table 4). For staff, perceived security risks, unstable housing, alleged/untrustworthiness by community leaders, and unreliable contact information/frequent travel often drove decisions not to offer the smartphone-based recording device to participants. Staff noted that although the device was generally easy to set up, usually requiring 5–10 minutes, participants unfamiliar with smartphones often struggled with basic functions like charging from the provided power bank. Upon returning the device, many participants also shared that they had felt discomfort about the visibility of the microphone in public settings. Research assistants suggested several improvements to support broader use, including real-time monitoring features and more discreet, wearable designs with fewer visible components.

**Table 4:** Qualitative experience of using cough recording devices, as observed by research staff.

Theme	Representative Quotes
The device was easy to use and set up	“Setting up the device can take like one minute but explaining the entire process… can take 5 to 8 minutes.” “It is just an app that was put on a smartphone… it starts immediately to do the recording.” “Setup is very easy… just having mobile data and logging into the Hyfe research app”
Participants had concerns about safety, stigma, and carrying the phone	“Some participants… were concerned people would think they were spying because of the microphone.” “My boyfriend thought I had another man because of the phone; I had to return it.” “Participants feared to go to the market or bar with the phone… it’s about security and privacy”
Participants saw value in the device but had mixed reactions	“Some said it was positive if the phone could give them feedback on their cough monitoring.” “Some were excited that the device could help diagnose TB without sputum.” “Participants saw it as improving their treatment or differentiating TB from normal cough”
Future improvements should focus on hardware, privacy, and RA support	“We need devices that don’t require microphones or internet.” “Make a smaller device like an earpiece that can record discreetly.” “Give RAs dashboard access so they can see data in real-time”

Note: Research staff reported participant feedback and experiences during device distribution and collection. The qualitative component included formal interviews with research staff only; these quotes do not represent direct participant interviews

## Discussion

This study aimed to assess the diagnostic performance and implementation feasibility of smartphone-based cough frequency recording for differentiating individuals with versus without TB in both community-based and health facility-based settings in Kampala, Uganda. While people with TB had significantly higher cough frequency than those without TB in both cohorts, cough frequency alone did not meet the 2014 WHO TPP benchmarks for TB screening (≥90% sensitivity and ≥70% specificity) [9] or the 2025 WHO TPP thresholds for high-sensitivity screening (Type 2) or high-specificity triage (Type 3) profiles [10]. Objectively measured cough frequency was only weakly correlated with subjectively reported cough presence, severity, and durations. In measuring cough frequency, staff encountered significant implementation challenges including participant concerns about device visibility and safety, which limited completion of cough recording. These findings suggest that, while recorded cough frequency may signal important TB disease-related differences and could add value as part of a broader diagnostic strategy, currently available recording apps are unlikely to be useful as standalone tests for TB.

Although cough frequency alone fell short of consensus accuracy targets, its association with TB status highlights its potential utility. Prior studies by Lee et al. and Turner et al. have demonstrated that objectively measured cough is a stronger predictor of TB disease severity than self-reported symptoms, with elevated cough linked to greater infectiousness [19,20]. These findings suggest that high cough frequency among individuals with TB may serve as an indicator of both clinical significance and transmission risk. However, elevated cough frequency is non-specific and may also reflect other non-infectious conditions such as asthma, gastroesophageal reflux disease, or smoking-related chronic cough, which limits its value as a standalone indicator of TB. Identifying individuals with elevated cough burdens, even in the absence of definitive diagnostic confirmation, is likely still essential for interrupting transmission. Diagnostic performance may also be enhanced with further integration of AI. AI-enabled tools that incorporate acoustic, spectral, and statistical features of cough sounds have shown promise in improving detection and classification of coughs [21–23]. Collectively, these findings support the potential role of smartphone-based cough monitoring as a complementary tool within multi-modal TB detection strategies. That said, both self-report and acoustic recording approaches are susceptible to errors including recall bias in self-reporting, misclassification of ambient sounds as cough, and misattribution of nearby persons’ coughs to the participant in acoustic monitoring, all of which can affect the accuracy of cough assessment.

The weak correlation of measured cough frequency with reported cough symptoms may reflect differences in the dimensions of cough captured by each measure, although there are also factors that limit the accuracy of each. Individuals with chronic respiratory conditions, allergies, or smoking history may perceive their cough as less severe, viewing it as a typical part of their baseline health rather than a sign of illness. Factors such as age, sex, concern about underlying disease, recall bias, and cultural norms may also affect how symptoms are reported. Thus, objective measures such as app-based recorded cough frequency over 48 hours may offer greater reliability. However, recorded cough frequency also faces several potential sources of error. Cough has been shown to vary from one day to the next, perhaps due to natural variation or to variability in activities and environmental allergen or irritant exposures. Our 48-hour cough monitoring period may therefore have been too short to accurately estimate each participant’s average cough frequency, and circadian variability in cough patterns may further affect the representativeness of any fixed monitoring period [24,25].

Implementing longitudinal cough recording in our urban Ugandan research setting posed substantial logistical challenges, resulting in a low proportion of participants with complete and usable data. Barriers such as limited smartphone familiarity, concerns about device visibility, and unstable housing conditions reduced both participant interest and staff willingness to distribute recorders consistently. These barriers made it more difficult to conduct extended cough monitoring. Future efforts could benefit from making devices smaller and less conspicuous, for example with wrist-worn sensors or pendant-like microphones, and from designing with extended battery life to reduce dependence on regular access to electricity. In addition, efforts to integrate real-time feedback mechanisms, enhance ambient noise filtering, or incorporate hybrid sensor approaches (e.g., combining acoustic and location data to increase noise filtering in known crowded locations) could improve recording accuracy in busy environments. Implementation in settings with higher levels of smartphone ownership and greater digital literacy may support longer recording periods and improve data completeness. Collectively, these adaptations could enhance the feasibility, acceptability and diagnostic value of cough monitoring as part of TB screening strategies. Future research should also consider longitudinal monitoring approaches in which changes from an individual’s personalized baseline cough frequency, rather than a single population-level threshold, are used to flag potential disease onset or progression.

Although this study offers novel insights on cough frequency in a TB screening context, there are some limitations to its accuracy estimates. First, participant sampling was uneven by initial Ultra results, requiring inverse probability weighting to more accurately reflect the underlying screening and diagnostic populations, and there may also have been small demographic differences introduced by matching that we did not account for. Second, completion rates were low due to operational barriers. This may have introduced selection bias, and in particular, the discretion exercised by study staff in offering devices may have led to underrepresentation of individuals with social and environmental risk factors such as unstable housing or crowded living conditions. Furthermore, in this study’s crowded urban environment, ambient noises may have been misclassified as cough or sounds of others coughing nearby may have been misattributed to participants. Although the use of lapel microphones aimed to improve the specificity of cough attribution, some measurement errors are likely to have remained. Third, TB status classification was based on microbiological results from expectorated sputum, using a combination of Ultra (in those without known prior TB) and culture testing. Inadequate sensitivity of expectorated sputum could have caused some people with TB to be misclassified as having indeterminate or negative TB status. This misclassification may have been greatest among those with fewer respiratory symptoms. A secondary analysis using culture alone as the reference standard yielded results consistent with the primary composite analysis, suggesting that the inclusion of Ultra in the reference standard did not meaningfully alter diagnostic accuracy estimates. Additionally, the qualitative component of this study captured the perspectives of research staff only and did not include direct interviews with study participants. The absence of participant voices represents a meaningful gap, as the experiences and preferences of potential end users are essential for informing human-centered device design. Lastly, the observation that self-reported cough had lower sensitivity among participants who completed cough monitoring than might be expected in the broader enrolled population suggests that willingness to participate in cough monitoring may have been associated with symptom experience, potentially introducing selection bias.

## Conclusion

Smartphone-based cough monitoring alone does not meet the 2014 WHO TPP threshold for TB screening [9], nor does it meet the 2025 WHO TPP thresholds for high-sensitivity screening (Type 2) or high-specificity triage (Type 3) tests [10]. It may nonetheless offer complementary information about respiratory pathology when integrated with existing risk factors and screening tools. The 2025 WHO TPP’s differentiated framework for different testing contexts [10] highlights that moderate-sensitivity tools could still contribute within two-step algorithms or resource-limited settings, though development and evaluation of such combinations would be needed to confirm this. Future development should prioritize human-centered redesign of monitoring devices, incorporating the perspectives of potential users in the design process, to improve acceptability, data completeness, and feasibility. Analytical methods for interpreting cough patterns, including personalized baseline monitoring, should also be developed to enhance diagnostic value in high-burden, resource-limited settings.

## Supporting information

S1 Checklist Inclusivity in global health research questionnaire. (DOCX)

S1 Text Supporting information. (DOCX)

## Abbreviations

AI: Artificial intelligence
AUC: Area under the curve
CI: Confidence interval
CXR: Chest x-ray
HIV: Human immunodeficiency virus
IQR: Interquartile range
ROC: Receiver operating characteristic
SGRQ: Saint George’s Respiratory Questionnaire
TB: Tuberculosis
TPP: Target product profile
WHO: World Health Organization
