# Supplementary material for: Evaluating smartphone-based cough frequency monitoring for tuberculosis screening and triage in Uganda: A mixed-methods evaluation
Source: PLOS Glob Public Health. 2026 Oct 1;6(10):e0005201. doi: 10.1371/journal.pgph.0005201 (PMC13630198; doi:10.1371/journal.pgph.0005201)
Supplement: Supplementary file 2 [file pgph.0005201.s002.docx]

**Supporting Information**

**Table A.** **Inverse probability weights used to account for sampling by enrollment location and Xpert Ultra result category.** Weights were calculated as the ratio of the proportion enrolled to the proportion tested within each stratum.

| **Enrollment location** | **Xpert Ultra result** | **Proportion among those tested (%)** | **Proportion among those enrolled (%)** | **Weight** |
| --- | --- | --- | --- | --- |
| Community | Negative | 99.0 | 35.3 | 0.98 |
|  | Trace | 0.4 | 35.3 | 0.004 |
|  | Positive | 0.6 | 29.4 | 0.006 |
| Health Facility | Negative | 91.4 | 30.6 | 0.91 |
|  | Trace | 0.9 | 42.1 | 0.006 |
|  | Positive | 7.7 | 27.3 | 0.085 |

**Table B. STARD checklist.** Standards for Reporting of Diagnostic Accuracy Studies for the present study.

|  | **Section & Topic** | **No** | **Item** | **Reported on page #** |
| --- | --- | --- | --- | --- |
|  |  |  |  |  |
|  | **TITLE OR ABSTRACT** |  |  |  |
|  |  | **1** | Identification as a study of diagnostic accuracy using at least one measure of accuracy  (such as sensitivity, specificity, predictive values, or AUC) | 1, 2 |
|  | **ABSTRACT** |  |  |  |
|  |  | **2** | Structured summary of study design, methods, results, and conclusions  (for specific guidance, see STARD for Abstracts) | 2 |
|  | **INTRODUCTION** |  |  |  |
|  |  | **3** | Scientific and clinical background, including the intended use and clinical role of the index test | 4-5 |
|  |  | **4** | Study objectives and hypotheses | 5 |
|  | **METHODS** |  |  |  |
|  | *Study design* | **5** | Whether data collection was planned before the index test and reference standard  were performed (prospective study) or after (retrospective study) | 5 |
|  | *Participants* | **6** | Eligibility criteria | 5-6 |
|  |  | **7** | On what basis potentially eligible participants were identified  (such as symptoms, results from previous tests, inclusion in registry) | 5-6 |
|  |  | **8** | Where and when potentially eligible participants were identified (setting, location and dates) | 5-6 |
|  |  | **9** | Whether participants formed a consecutive, random or convenience series | 5-6 |
|  | *Test methods* | **10a** | Index test, in sufficient detail to allow replication | 6-7 |
|  |  | **10b** | Reference standard, in sufficient detail to allow replication | 7-8 |
|  |  | **11** | Rationale for choosing the reference standard (if alternatives exist) | 7-8 |
|  |  | **12a** | Definition of and rationale for test positivity cut-offs or result categories  of the index test, distinguishing pre-specified from exploratory | 8 |
|  |  | **12b** | Definition of and rationale for test positivity cut-offs or result categories  of the reference standard, distinguishing pre-specified from exploratory | 7-8 |
|  |  | **13a** | Whether clinical information and reference standard results were available  to the performers/readers of the index test | 6-7 |
|  |  | **13b** | Whether clinical information and index test results were available  to the assessors of the reference standard | 7-8 |
|  | *Analysis* | **14** | Methods for estimating or comparing measures of diagnostic accuracy | 8-10 |
|  |  | **15** | How indeterminate index test or reference standard results were handled | 8, 11 |
|  |  | **16** | How missing data on the index test and reference standard were handled | 11 |
|  |  | **17** | Any analyses of variability in diagnostic accuracy, distinguishing pre-specified from exploratory | 9-10, 13 |
|  |  | **18** | Intended sample size and how it was determined | 5-6 |
|  | **RESULTS** |  |  |  |
|  | *Participants* | **19** | Flow of participants, using a diagram | 10 |
|  |  | **20** | Baseline demographic and clinical characteristics of participants | 11-12 |
|  |  | **21a** | Distribution of severity of disease in those with the target condition | 11-12 |
|  |  | **21b** | Distribution of alternative diagnoses in those without the target condition | 11-12 |
|  |  | **22** | Time interval and any clinical interventions between index test and reference standard | 6, 11 |
|  | *Test results* | **23** | Cross tabulation of the index test results (or their distribution)  by the results of the reference standard | 12-13 |
|  |  | **24** | Estimates of diagnostic accuracy and their precision (such as 95% confidence intervals) | 12-13 |
|  |  | **25** | Any adverse events from performing the index test or the reference standard | N/A |
|  | **DISCUSSION** |  |  |  |
|  |  | **26** | Study limitations, including sources of potential bias, statistical uncertainty, and generalisability |  |
|  |  | **27** | Implications for practice, including the intended use and clinical role of the index test | 19-20 |
|  | **OTHER INFORMATION** |  |  |  |
|  |  | **28** | Registration number and name of registry | N/A |
|  |  | **29** | Where the full study protocol can be accessed | N/A |
|  |  | **30** | Sources of funding and other support; role of funders | N/A |
|  |  |  |  |  |

**Appendix A. Symptom questionnaires**. Symptom questionnaire administered during enrollment to participants in the health facility cohort and in the community cohort.

Symptom questionnaire during enrollment (for health facility cohort)

1. Which of the following symptoms do you have currently? (Currently can mean today or within the past few days.)
   Select all that apply:

- Cough
- Coughing up blood
- Unexplained fever or chills
- Unexplained fatigue
- Drenching sweats at night
- Shortness of breath
- Pain my chest
- Loss of normal appetite (anorexia)
- None of the above
- Unknown/refused.

3. You said that you currently have a cough. Now looking back in time, for how long have you had this cough?
   (Interviewer should record answer in weeks.)
4. Use the visual scale below to indicate the severity of the cough in past week:
   a.

5. Number of coughs during a 5-minute observation period (as observed by person administering the CRF)
6. To be answered by the RA: How much did the participant cough during this interview?
   a. Not at all
   b. Very little (e.g. mild cough one or two times)
   c. Occasionally (e.g. slight cough 3-5 times, or 1-2 moderate coughing episodes
   d. Frequently (e.g. a frequent light cough, or 3-5 moderate coughing episodes")
   e. Persistently/severely.

Symptom questionnaire during screening (for community cohort)

1. TB sometimes has no symptoms, so we are testing everyone for TB regardless of whether or not they have symptoms. But we would like to know what symptoms you currently have. Do you have cough?
7. (If yes) For how many days have you had cough? Use the visual scale below to indicate the severity of the cough in past week:
   a.

8. Number of coughs during a 5-minute observation period (as observed by person administering the CRF)
9. To be answered by the RA: How much did the participant cough during this interview?
   a. Not at all
   b. Very little (e.g. mild cough one or two times)
   c. Occasionally (e.g. slight cough 3-5 times, or 1-2 moderate coughing episodes
   d. Frequently (e.g. a frequent light cough, or 3-5 moderate coughing episodes")
   a. Persistently/severely.

**Appendix B. Staff interview questions**. Interview questions asked during staff interviews.

1. Can you describe how you explained the cough recording device to participants?
2. How would you describe the ease of use (in respect to the setup) of the device?
3. How long (in minutes) did it usually take to set up and explain the device to participants?
4. Can you describe any negative feedback you received from participants?
5. Can you describe any positive feedback you received from participants?
6. What factors play into your decision not to give the device to a participant?
7. How can the device/software be improved for future studies?
8. How can future studies be improved to better support RAs/staff in implementing cough recorders?
9. Any other feedback you would like to share with the team?

**Table C. Cough thresholds to meet WHO TPP profiles**

| **TPP Profile** | **Required Sensitivity** | **Required Specificity** | **Cohort** | **CPH Threshold (95% CI)** | **Sensitivity (95% CI)** | **Specificity (95% CI)** | **Meets Sens. Target** | **Meets Spec. Target** |
| --- | --- | --- | --- | --- | --- | --- | --- | --- |
| 2014 WHO TPP | ≥90% | ≥70% | Community | 0.3 (0.2–0.7) | 89.9% (88.7–91.3%) | 16.3% (7.8–52%) | No | No |
|  | ≥90% | ≥70% | Health Facility | 2.5 (0.2–3.4) | 91.2% (87.3–92.8%) | 52.2% (0–71.7%) | Yes | No |
| 2025 Type 2 – minimal | ≥90% | ≥60% | Community | 0.3 (0.2–0.7) | 89.9% (88.7–91.3%) | 16.3% (7.8–52%) | No | No |
|  | ≥90% | ≥60% | Health Facility | 2.5 (0.2–3.4) | 91.2% (87.3–92.8%) | 52.2% (0–71.7%) | Yes | No |
| 2025 Type 2 – optimal | ≥95% | ≥85% | Community | 0.2 (0.1–0.6) | 95% (93.8–95.8%) | 16.3% (2.2–37.8%) | No | No |
|  | ≥95% | ≥85% | Health Facility | 0.9 (0.2–2.6) | 93.9% (91.9–97.7%) | 28.3% (0–64.7%) | No | No |
| 2025 Type 3 – minimal | ≥60% | ≥98% | Community | 6.4 (4.7–6.4) | 21.7% (10.8–38.1%) | 98% (97.5–98.3%) | No | No |
|  | ≥60% | ≥98% | Health Facility | 33.7 (12.3–33.7) | 16.9% (4.4–59%) | 97.8% (97.2–98.2%) | No | No |
| *CPH: coughs per hour. Threshold is the minimum CPH value at which a participant is classified as TB-positive. For 2014 WHO TPP and 2025 Type 2 profiles, the threshold is fixed at the sensitivity target. For 2025 Type 3 profiles, the threshold is fixed at the specificity target. 95% CIs derived from 1,000 bootstrap resamples.* | | | | | | | | |
